# The Effectiveness of a Mechanical Ventilation System for Indoor PM_2.5_ in Residential Houses

**DOI:** 10.3390/toxics11110912

**Published:** 2023-11-07

**Authors:** Dongho Shin, Younghun Kim, Kee-Jung Hong, Gunhee Lee, Inyong Park, Hak-Joon Kim, Sangwoo Kim, Cheong-Ha Hwang, Kwang-Chul Noh, Bangwoo Han

**Affiliations:** 1 Department of Sustainable Environment Research, Korea Institute of Machinery & Materials, Deajeon 34103, Republic of Korea; sdh302@kimm.re.kr (D.S.); diayolk@kimm.re.kr (H.-J.K.); 2 Department of Mechanical Engineering, Yonsei University, Seoul 03722, Republic of Korea; 3 Air Lab, Inc., Gwangju 62278, Republic of Koreacreative@c-airlab.com (K.-C.N.)

**Keywords:** mechanical ventilation, CADR, PM_2.5_, MERV, residential house

## Abstract

The mechanical ventilation systems used in houses are designed to reduce carbon dioxide emissions while minimizing the energy loss resulting from ventilation. However, the increase in indoor fine particulate (PM_2.5_) concentration because of external PM_2.5_ influx through the ventilation system poses a problem. Here, we analyzed the changes in indoor PM_2.5_ concentration, distinguishing between cases of high and low outdoor PM_2.5_ concentrations and considering the efficiency of the filters used in residential mechanical ventilation systems. When using filters with the minimum efficiency reporting value (MERV) of 10 in the ventilation system, the outdoor PM_2.5_ concentration was 5 μg/m³; compared to the initial concentration, the indoor PM_2.5_ concentration after 60 min decreased to 73%. When the outdoor PM_2.5_ concentration was 30–40 μg/m³, the indoor PM_2.5_ concentration reached 91%. However, when MERV 13 filters were used, the indoor PM_2.5_ concentration consistently dropped to 73–76%, regardless of the outdoor PM_2.5_ concentration. Furthermore, by comparing the established equation with the mass balance model, the error was confirmed to be within 5%, indicating a good fit. This allows for the prediction of indoor PM_2.5_ under various conditions when using mechanical ventilation systems, enabling the formulation of strategies for maintaining indoor PM_2.5_, as recommended by the World Health Organization.

## 1. Introduction

People spend a significant proportion of their time indoors [1,2,3]. This includes the time spent in residences, where they sleep and eat, and in workplaces. Commuting using public transportation or vehicles to reach the workplace is a substantial indoor activity. Thus, a major portion of time is dedicated to being indoors.

The primary pollutants originating from indoor environments encompass a wide range of sources. These include airborne microorganisms from humidifiers, air conditioning units, refrigerators, pets, and food waste [4,5]. Furthermore, formaldehyde is emitted by furniture, insulating materials, and plywood [6,7,8]. Moreover, acetone is emitted from synthetic resins and adhesives, and combustion gases (carbon monoxide and oxides of nitrogen and sulfur) are emitted from stoves and gas ranges [9,10]. Radon emitted from latex matrices and building materials [11] along with volatile organic compounds from cigarette smoke and fine particulate matter are also prevalent indoor contaminants [12,13,14].

Prolonged exposure to these pollutants can lead to skin conditions, respiratory illnesses, and even lung cancer [15]. Furthermore, recent research has suggested that fine particulate matter can affect the stomach and cause colorectal cancer [16]. However, the recognition of indoor pollutants is often challenging. One method for mitigating indoor pollution is the frequent ventilation of indoor spaces via opening windows. Nonetheless, on days with high outdoor particulate matter levels, natural ventilation may increase indoor particulate matter concentrations, which could lead to energy losses during summer and winter owing to heating or cooling [17,18]. Consequently, mechanical ventilation systems are required, and extensive research is being conducted on managing indoor air quality using such systems.

Mechanical ventilation systems are designed to bring in outdoor air and expel indoor air, utilizing the heat exchange between indoor and outdoor air to minimize heating and cooling losses. In addition, these systems are equipped with filters to prevent the influx of fine particulate matter and ensure a clean air supply [19]. Starting in 2006, South Korea introduced mandatory ventilation systems in residential complexes with more than 100 units [20]. Consequently, many apartment buildings are equipped with mechanical ventilation systems. Filter efficiency regulations based on outdoor air intake have also been strengthened. Currently, the ventilation systems in residential complexes and multi-use facilities are required to achieve a capture efficiency of over 60% using particle-counting methods. While residential complexes have a specified minimum ventilation rate of at least 0.5 air change per hour, multi-use facilities regulate ventilation based on per-person ventilation rate.

Various studies have defined the factors that influence indoor air quality and assessed their impacts. Noh and Yook (2016) evaluated the effectiveness of air purifiers and their circulation effects in university lecture rooms [21]. Martins and da Graca (2017) reported that outdoor fine particulate matter (PM_2.5_), which infiltrates indoors via natural ventilation, is the primary source of indoor PM_2.5_ [22]. Ben-David and Waring (2016) simulated the impacts of natural and mechanical ventilation on indoor pollutant concentrations and energy usage in office buildings and proposed ventilation strategies [23]. Ruan and Rim (2019) measured indoor PM_2.5_ and ozone concentrations based on the air handling unit and occlusion-aware filter efficiency in offices and analyzed the effects of filter efficiency and outdoor air concentration on indoor PM_2.5_ concentrations [24]. They also conducted an analysis on the influence of ventilation rates and filter efficiency on the indoor PM_2.5_ and ozone concentrations in office buildings. Despite the diverse range of studies on pollutant control via mechanical ventilation, most have focused on ventilation devices in office spaces [25,26], while studies analyzing the effects of ventilation systems in actual residential houses are limited. In the case of offices, the frequent influx and outflow of people through entry points make it challenging to predict changes over time. Additionally, a higher number of occupants per unit area necessitates a higher ventilation rate. However, in residential houses, the lower occupancy per unit area results in a reduced demand for ventilation, leading to lower airflow requirements for mechanical ventilation systems. Thus, it is easier to predict concentration changes over time using numerical models. For these reasons, while previous research has focused on comparing the effectiveness of filters based on indoor concentrations that converge when using ventilation system filters, our study expressed the effectiveness of mechanical ventilation in terms of Clean Air Delivery Rate (CADR) to determine whether ventilation systems can effectively and rapidly reduce indoor PM_2.5_ concentrations.

In this study, indoor concentrations based on filter efficiency were measured in residential mechanical ventilation systems. Variations in indoor PM_2.5_ concentrations were analyzed between periods of high and low outdoor PM_2.5_ concentrations. Furthermore, a mass balance model was formulated to compare the actual measurements with theoretical values, deriving an equation that accurately predicted the indoor PM_2.5_ concentrations. The accuracy of this equation was verified not only in the experimental houses, but also in various other residential houses. This study thus devised an approach to predicting indoor PM_2.5_ concentrations using mechanical ventilation systems based on outdoor PM_2.5_ concentrations and ventilation system filter grades. This approach can be used to maintain indoor PM_2.5_ concentrations below 10 µg/m³.

## 2. Materials and Methods

**Figure 1:**
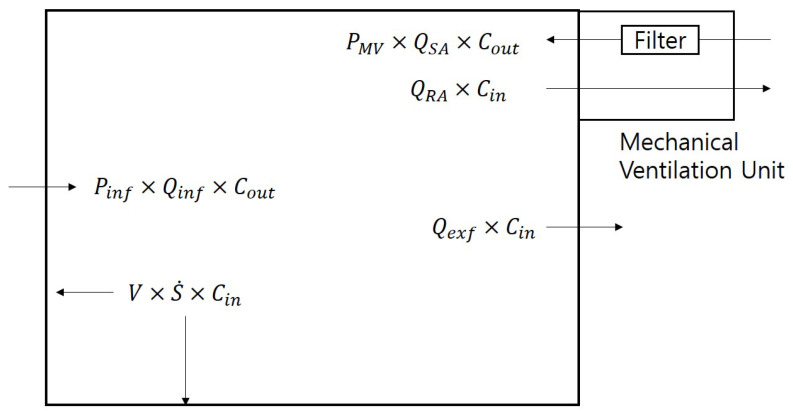
Schematic diagram of indoor particle concentration with a mechanical ventilation system.

Figure 1 depicts a schematic that models the factors affecting indoor particle concentrations when a ventilation system is used in an actual residential house.

The factors that influence the indoor concentrations applied in the model include the particles entering and exiting via the ventilation system, those entering and exiting via the building envelope, and particles settling naturally. Through the modeling process, an equation describing the variation in indoor concentrations over time was formulated as follows:

(1) $$V\frac{dC_{in}(t)}{dt}=P_{MV}\times Q_{SA}\times C_{out}(t)-Q_{RA}\times C_{in}(t)+P_{inf}\times Q_{inf}\times C_{out}(t)-Q_{exf}\times C_{in}(t)-V\times \dot{S}\times C_{in}(t)$$

Here, $C_{in}$ represents the indoor PM_2.5_ concentration, V is the volume of the interpreted space, $Q_{SA}$ is the indoor supply airflow rate due to the ventilation system, $P_{MV}$ is the particle penetration coefficient of the ventilation system filter, $P_{inf}$ is the particle penetration coefficient through the apartment envelope, $Q_{inf}$ is the airflow rate entering through the apartment envelope, $C_{out}$ is the outdoor PM_2.5_ concentration, $Q_{exf}$ is the airflow rate exiting the indoor space through the apartment envelope, and $\dot{S}$ is the deposition rate by settling. Solving the differential equation in Equation (1) leads to the following Equation (2):

(2) $$C_{in}\left(t_{i}\right)=\left(C_{in}(t_{i-1})-\frac{P_{MV}\times Q_{SA}+P_{inf}\times Q_{inf}}{Q_{RA}+Q_{exf}+V\times \dot{S}}\times C_{out}(t_{i-1})\right)\times exp\left(-\frac{Q_{RA}+Q_{exf}+V\times \dot{S}}{V}(t_{i}-t_{i-1})\right)+\frac{P_{MV}\times Q_{SA}+P_{inf}\times Q_{inf}}{Q_{RA}+Q_{exf}+V\times \dot{S}}\times C_{out}(t_{i-1})$$

Equation (2) represents the indoor PM_2.5_ concentration over time as an exponential function of its relationship with the outdoor PM_2.5_ concentration. In this study, the current indoor PM_2.5_ concentration ($C_{in}\left(t_{i}\right)$) was influenced by the prior indoor PM_2.5_ concentration ($C_{in}\left(t_{i-1}\right)$) and the inflow of the prior outdoor PM_2.5_ concentration ($C_{out}\left(t_{i-1}\right)$), and the real-time outdoor PM_2.5_ concentration was measured and applied at one minute intervals for calculation.

**Figure 2:**
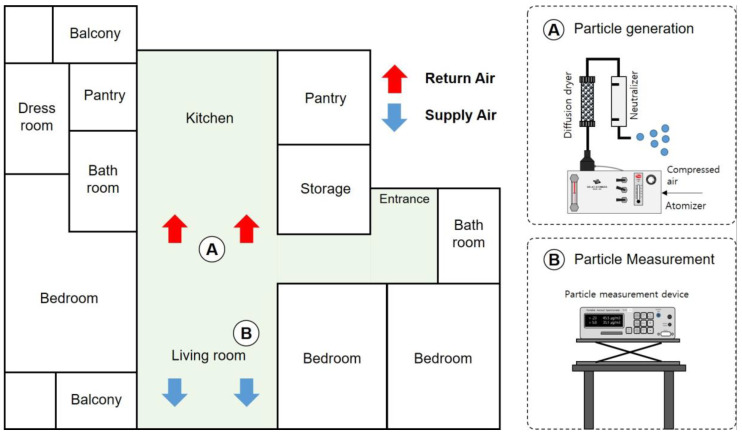
Floor plan of the test house and experimental setup. A is the particle generation system and B is the particle measurement system.

Figure 2 shows the experimental setup used to investigate the changes in the indoor particle concentrations using ventilation systems in a residential house. The experiment was conducted in an apartment built in 2018 with a dedicated area of 72 m². Only the kitchen and living room areas were utilized for the experiment, with a calculated volume of 84 m^3^. Particle measurements were performed at location B using an optical particle counter (1.109, Grimm Aerosol Technik, Ainring, Germany) placed on a table in the living room. The test particles were generated at location A using a potassium chloride 1% solution in a six-jet atomizer (9306, TSI, Shoreview, MN, USA), which was passed through a diffusion dryer to remove moisture and neutralized using a neutralizer (3012, TSI) with a krypton-85 source. The generated KCl particles had a monodisperse mass distribution as a function of particle size; their mass median diameter was 0.3 µm and their geometric standard deviation was about 1.4. A ventilation system was installed on the exterior side of the living room ceiling with two supply and exhaust diffusers connected to the living room and kitchen. The flow rate of the diffusers was measured using a flowmeter (6750, KANOMAX, Osaka, Japan), revealing a combined supply flow rate of 44 m³/h and an exhaust flow rate of 56 m³/h. To determine the infiltration and exfiltration flow rates through the apartment envelope, airtightness measurements were conducted following standardized tests (EN13829 [27] and ASTM E779-10 [28]) and compared with the decay of carbon dioxide (CO_2_) concentrations under normal temperature and pressure conditions. The CO_2_ concentration was measured with an IAQ sensor (IQ 610, GrayWolf Sensing Solutions, Shelton, CT, USA).

In this study, the *CADR*, a metric commonly used to indicate air purifier performance, was used to quantify the particle removal efficiency of the ventilation system. *CADR* represents the volume of clean air delivered by an air purifier per unit of time and is calculated by multiplying the airflow rate of the air purifier by the particle collection efficiency of the filter. Similarly, ventilation systems supply clean air by filtering out pollutants from the incoming outdoor air. Therefore, the particle reduction effect of the ventilation system can be expressed using the *CADR* ventilation system. It is defined by the following equation:

(3) $${CADR}_{MV}=V\times {\left.\left(\frac{lnC_{2}-lnC_{1}}{t_{2}-t_{1}}\right)\right|}_{on}-V\times {\left.\left(\frac{lnC_{2}-lnC_{1}}{t_{2}-t_{1}}\right)\right|}_{off}={CADR}_{MV,on}-{CADR}_{MV,off}$$

The *CADR*, as defined by the standard test protocol (SPS-KACA002-132 [29]), is calculated as the product of the slope of the indoor concentration decay curve over time and the experimental space volume. However, in actual residential houses, indoor particle concentrations often exhibit a decay pattern in the form of *C_in_*(*t*) = *exp*(−*kt*) + *A*, rather than a simple exponential decay of *C_in_*(*t*) = *exp* (−*kt*). Therefore, defining the CADR in an actual environment requires adopting a formulation similar to the aforementioned pattern. In this study, *t*_1_ for the *CADR* via mechanical ventilation (*CADR_MV_*) was considered to be approximately 2–3 min after the operation of the ventilation system, and *t*_2_ was set to 20 min after *t*_1_. According to the standard testing protocol SPS-KACA002-132, more than 20 measurement points or measurements should be marked until the time is equal to 1/10 of the initial particle concentration. Hence, the duration *t*_2_−*t*_1_ was set to 20 min. Using Equation (2) for calculations and fitting, the results indicated that, when the time exceeded 40 min, the R^2^ value decreased below 0.99. Based on this observation, Equation (3) was utilized to calculate the *CADR_MV_* and accurately define the purification capability of the ventilation system.

In this study, filters with minimum efficiency reporting value (MERV) ratings of 10 and 13 were used and compared. Following the standard testing protocol SPS-KACA002-132, tests were conducted to determine the fine particle removal efficiencies of the filters. The PM_2.5_ removal efficiency of the MERV 10 filter was approximately 7%, whereas the MERV 13 filter exhibited an efficiency of around 90%. The particle reduction effects originating from the ventilation system were examined by comparing the differences in particle capture efficiency between these filters.

## 3. Results

**Figure 3:**
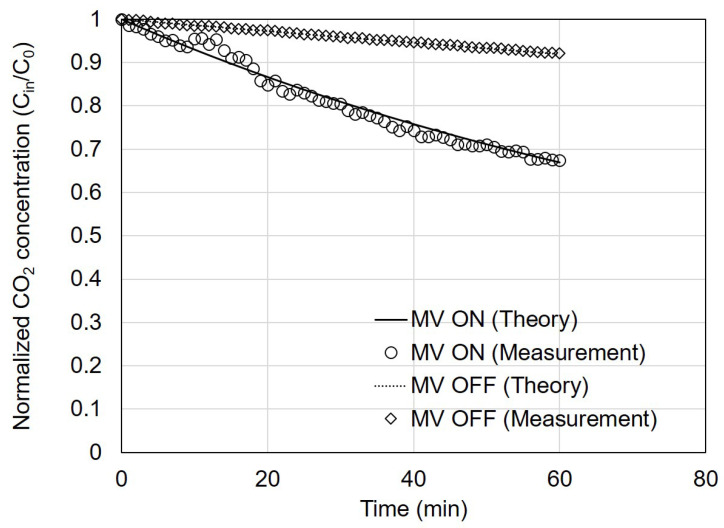
Normalized carbon dioxide (CO_2_) concentrations with operation of a mechanical ventilation system on and off according to elapsed time. The marked symbols are the measured concentrations and the lines represent the estimates from the theoretical calculation.

Figure 3 shows the measured changes in CO_2_ concentration over time with and without the ventilation system after introducing CO_2_ into the test house. A comparison was made between the results calculated using Equation (2) and the actual measured data.

First, the measured and theoretical values were compared without the ventilation system operating. The air leakage rate of the test house, as measured using the building envelope standard test, showed an ACH50 value of 2.1 air changes per hour (ACH). This value can be converted into ACH under atmospheric pressure conditions by dividing ACH50 by 20 [30], resulting in an ACH of 0.11 for the test house. Using this, the calculated value of $Q_{inf}$ was 0.13 m³/h. Applying this value to Equation (2), the calculated MV OFF value was determined to be 92% after 60 min. The measured value of MV OFF exhibited a similar trend, reaching 92% after 60 min with an error within 0.5%. 

Second, the measured and theoretical values were compared with the ventilation system operating. The airflow rates of the supply and exhaust diffusers were measured to be 44 and 56 m³/h, respectively. These airflow rates were input to Equation (2) for calculation and comparison with the measured data. The measured PM_2.5_ concentrations after 60 min decreased to approximately 67% of the initial concentration, which was consistent with the theoretical value.

**Figure 4:**
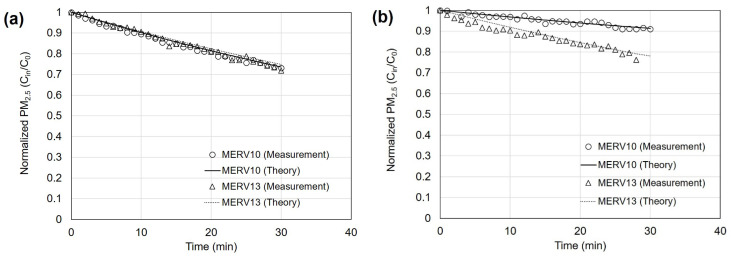
Normalized PM_2.5_ concentrations with filters with minimum efficiency reporting values (MERV) of 10 and 13 installed in the mechanical ventilation system according to elapsed time. Outdoor PM_2.5_ concentrations are in the ranges of (**a**) 0–5 μg/m³ and (**b**) 30–40 μg/m³. Marked symbols are measured concentrations and lines represent estimation ones from the theoretical calculation.

Figure 4 shows graphs illustrating the variations in indoor PM_2.5_ concentration over time. The graphs compare the reduction levels based on the rating of the ventilation system filter and thus compare the measured values with those calculated using Equation (2). The ventilation system used for comparison the employed filters with MERV ratings of 10 and 13, representing low- and high-performance filters, respectively.

Figure 4a shows the measurements taken on days when the outdoor PM_2.5_ concentration was below 5 μg/m³. The initial indoor PM_2.5_ concentration was set to 47 μg/m³, and the ventilation system was operated for a total of 60 min. The results showed that, when using the MERV 13 filters, the PM_2.5_ concentration decreased to 72% of the initial value, whereas, with the MERV 10 filters, the concentration decreased to 73%. This indicated that the filter performance did not significantly impact the indoor PM_2.5_ concentration when the outdoor PM_2.5_ concentration was sufficiently low.

Figure 4b shows the effect of indoor PM_2.5_ concentration reduction over time by operating the ventilation system on days when the outdoor PM_2.5_ concentration ranged from 30 to 40 μg/m³. Using the relatively high-efficiency MERV 13 filter, the concentration after 60 min decreased to 76% of the initial value. On the other hand, with the relatively low-efficiency MERV 10 filters, the concentration decreased to approximately 91% of the initial value. This indicated that, when the outdoor PM_2.5_ concentration was higher, the dust particle concentration in the air supplied through the ventilation system increased, significantly influencing the indoor PM_2.5_ concentration reduction capability. Furthermore, by comparing the calculated results from Equation (2) with the measured values, the error was found to be within 1%. Ruan and Rim [24] also found that, compared to lower-efficiency filters, higher-efficiency mechanical ventilation filters can decrease the indoor PM_2.5_ concentrations in office spaces. However, the indoor concentration variations measured by Ruan and Rim [24] make it challenging to accurately predict indoor PM_2.5_ concentrations, which are approximately 25% of outdoor PM_2.5_ concentrations. This difficulty arises because, in office environments, unlike residential houses, people move freely, introducing factors beyond the inflow and outflow of air through ventilation systems. Unlike offices, houses offer easier control of variables, reducing such errors and enabling relatively precise predictions of indoor concentrations based on outdoor PM_2.5_ concentrations.

**Figure 5:**
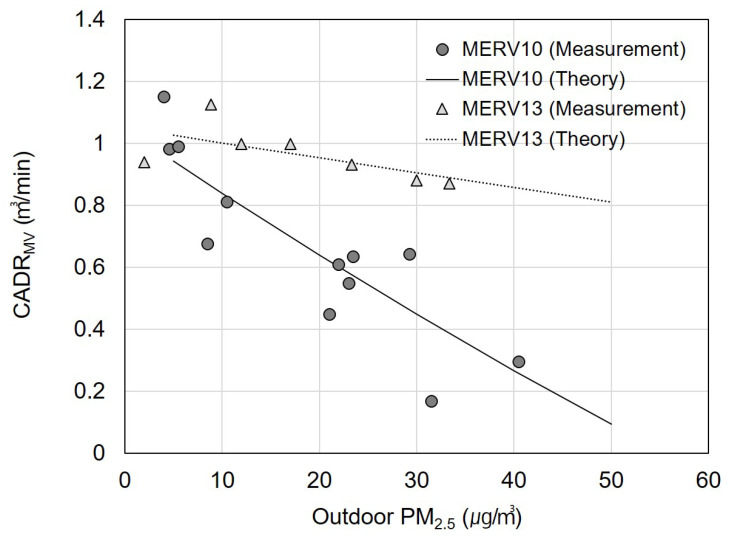
Experimental and theoretical clean air delivery rate for mechanical ventilation (*CADR_MV_*) with filters with minimum efficiency reporting values (MERV) of 10 and 13 installed in the mechanical ventilation system according to outdoor fine particulate matter (PM_2.5_) concentrations.

Figure 5 shows the *CADR_MV_* according to the outdoor PM_2.5_ concentrations. The measurements were conducted via alternating the application of the MERV 10 and 13 filters to the ventilation system while varying the outdoor PM_2.5_ concentration. The goal of these experiments was to analyze the indoor fine particle removal efficiency of the ventilation system based on the outdoor PM_2.5_ and filter ratings. Twelve measurements were performed using the MERV 10 filters and seven using the MERV 13 filters. In the figure, the circular data points represent the actual measured values and the lines indicate the *CADR_MV_* values calculated using the theoretical equation. The measured and calculated values were evidently in good agreement. Furthermore, on days when the outdoor PM_2.5_ was below 5 μg/m³, the *CADR_MV_* ranged from 0.9 to 1.2 m³/min, regardless of the filter grade. However, as the outdoor PM_2.5_ concentration increased, the *CADR_MV_* decreased, and the decrease rate varied based on the filter grade. When using the MERV 10 filters, at an outdoor PM_2.5_ concentration of 50 μg/m³ (considered to be an “unhealthy” air quality), the *CADR_MV_* decreased to 0.09 m³/min, which was ten times lower compared to 0.94 m³/min at 5 μg/m³. With the MERV 13 filters, at an outdoor PM_2.5_ concentration of 50 μg/m³, the *CADR_MV_* decreased to 0.81 m³/min, which was 1.3 times lower than 1.03 m³/min at 5 μg/m³. This demonstrated that the filter efficiency significantly affected the air purification capability of the ventilation system.

**Figure 6:**
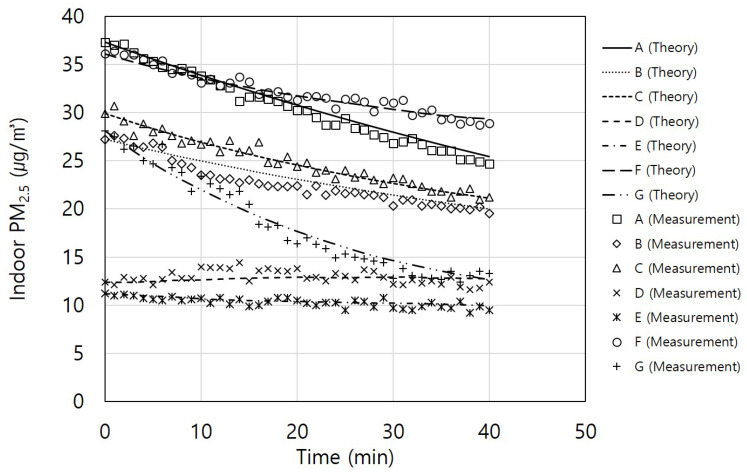
Indoor fine particulate matter (PM_2.5_) concentration changes in actual residential houses according to time.

Figure 6 presents the comparison between the measured indoor PM_2.5_ concentrations and those calculated using Equation (2) for seven residential houses (apartments) constructed between 2013 and 2018. The experiments were conducted by opening the windows of each apartment to allow outdoor air to enter and equilibrate with the outdoor PM_2.5_. Subsequently, the windows were closed and the ventilation systems were operated to measure the reduction in indoor PM_2.5_ concentrations.

Table 1 provides information on the construction year, floor area, test volume, ventilation system supply and exhaust airflow rates, filter efficiency, and other parameters used in the calculations. The ACH50, initial particle concentration, outdoor PM_2.5_ concentration, deposition rate, particle penetration through walls, and other relevant values used in Equation (2) are also included. The window frames in all apartments constructed within the last 10 years were made of polyvinyl chloride. For all apartments, the ACH50 value of 2.1/h measured from Apartment A was applied because their construction years were similar. When the construction year was similar, the air leakage rate tended to be comparable [31].

Atmospheric particles were used as test particles, and indoor and outdoor PM_2.5_ concentrations were simultaneously measured and incorporated into the equation. This study examined the use of various ventilation systems with different performance levels for different house types. When applying the equation established in this study, the degree of reduction in indoor PM_2.5_ concentrations was observed to vary. In particular, for houses D and E with low-efficiency filters (under 40%), a phenomenon was observed where, over time, the indoor PM_2.5_ concentration became similar to or even higher than the outdoor PM_2.5_ concentration when the initial concentration was lower than that outdoors. The equation aligned well with the observed data, particularly in cases where different ventilation system performances and filter efficiencies were applied. The filter performance when using the ventilation system was confirmed to have the most significant impact on indoor PM_2.5_ concentrations.

## 4. Discussion

A measurement analysis of the indoor concentration reduction achieved using ventilation systems in apartments was performed and represented using mathematical equations for comparison. Through previous experiments, this equation was verified to closely approximate the actual measured values. In the future, utilizing this equation will allow for the prediction of indoor fine PM_2.5_ concentrations during ventilation system operation based on outdoor PM_2.5_. This could help to develop methods for operating ventilation systems to reduce indoor exposure to fine particulate matter.

The impact of filter efficiency on indoor PM_2.5_ concentrations during ventilation system operating was confirmed. The use of different MERV-rated filters resulted in different pressure differences and airflow rates within the ventilation systems. Higher pressure differences led to reduced airflow rates, subsequently lowering the frequency of the indoor air exchange. Equation (2) was employed to analyze the reduction in CO_2_ and PM_2.5_ concentrations based on filter efficiency. An analysis was conducted for different outdoor PM_2.5_ concentration levels: good, moderate, and unhealthy.

**Figure 7:**
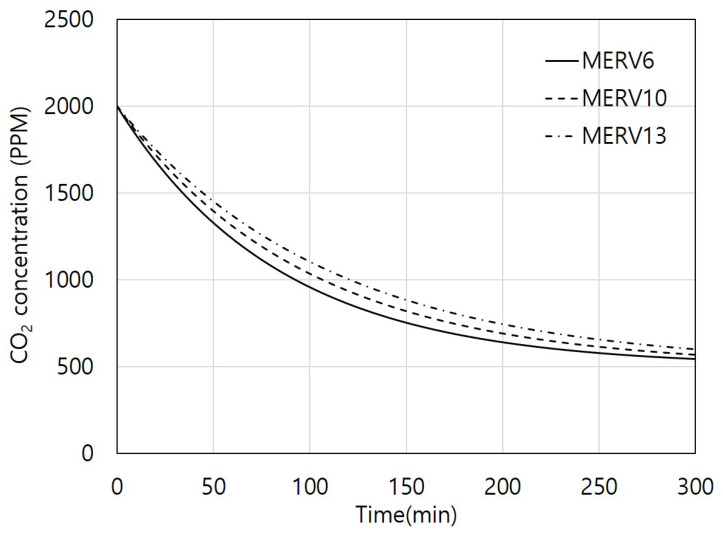
Changing indoor carbon dioxide (CO_2_) concentration according to time when using minimum efficiency reporting value (MERV) 6, 10, and 13 filters.

Figure 7 shows the calculated CO_2_ concentration over time using Equation (2) while considering three different filter grades and varying airflow rates resulting from filter pressure differences. MERV 6, 10, and 13 filters with airflow rates of 52, 44, and 39 m³/h, respectively, were utilized. The initial CO_2_ concentration was set at 2000 ppm, and the simulation was conducted for 300 min of ventilation system operation. The times required to reach the indoor air quality standard of 1000 ppm or less were 93, 107, and 121 min for the MERV 6, 10, and 13 filters, respectively. As the filter grade of the ventilation system increased, it led to higher pressure differences and reduced airflow rates. This can result in a lower CO_2_ removal capacity of the ventilation system.

**Figure 8:**
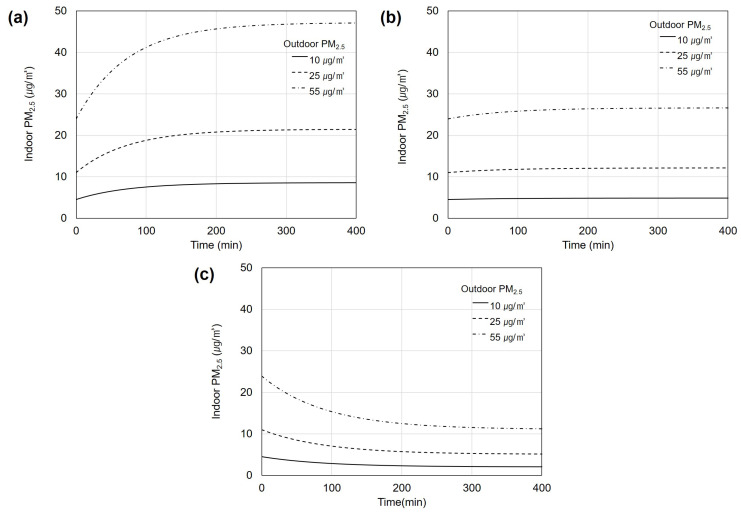
Indoor fine particulate matter (PM_2.5_) concentrations with different outdoor PM_2.5_ concentrations according to time. (**a**) Minimum efficiency reporting value (MERV) 6, (**b**) MERV 10, and (**c**) MERV 13 filters.

Figure 8 shows the changes in indoor PM_2.5_ concentration over a 400-min period using different MERV filter grades when the outdoor PM_2.5_ concentration was classified as “good”, “moderate”, or “unhealthy”. Figure 8a shows the indoor PM_2.5_ concentration when using the MERV 6 filter. The initial indoor PM_2.5_ concentration was assumed to be half of the outdoor PM2.5 concentration [32]. The efficiency of the MERV 6 filter for PM_2.5_ was set to 10%. When the outdoor PM_2_._5_ concentration was at the “good” level of 10 μg/m³, the initial indoor PM_2.5_ concentration was 4.5 μg/m³, increasing over time to reach 8.6 μg/m³ in the saturated state. When the outdoor PM_2.5_ concentration was at the “moderate” level of 25 μg/m³, the initial indoor PM_2.5_ concentration was 11 μg/m³, increasing to 19.7 μg/m³ in the saturated state. When the outdoor PM_2.5_ concentration was at the “unhealthy” level of 55 μg/m³, the initial indoor PM_2.5_ concentration was 23.9 μg/m³, increasing to 43.3 μg/m³ in the saturated state. Thus, using a low-efficiency filter in a ventilation system can increase the indoor PM_2.5_ concentration.

Figure 8b shows the indoor PM_2.5_ concentration when using the MERV 10 filter with a filter efficiency of 50%. After 400 min, after reaching the saturated state, the indoor PM_2.5_ concentration was 4.8 μg/m³ for outdoor PM_2.5_ concentrations of 10 μg/m³, 12.1 μg/m³ for 25 μg/m³, and 26.6 μg/m³ for 55 μg/m³. Overall, the use of the MERV 10 filter tended to maintain or slightly increase the initial PM_2.5_ concentration.

Figure 8c shows the indoor PM_2.5_ concentration when using the MERV 13 filter with a filter efficiency of 97%. After 400 min, after reaching the saturated state, the indoor PM_2.5_ concentration was 2.0 μg/m³ for an outdoor PM_2.5_ concentration of 10 μg/m³, 5.1 μg/m³ for 25 μg/m³, and 11.2 μg/m³ for 55 μg/m³. Overall, the indoor PM_2.5_ concentration decreased when using the MERV 13 filter.

These findings showed the importance of selecting a relatively high-efficiency filter for a ventilation system, as well as a ventilation rate that meets the regulatory ventilation requirements. This combination of enough ventilation and high-efficiency filtration is conclusively essential for maintaining indoor PM_2_._5_ concentrations of 10 μg/m³.

## 5. Conclusions

In the context of using mechanical ventilation systems for indoor CO_2_ reduction in houses, the impact of ventilation system filter efficiency on indoor PM_2.5_ concentrations was investigated, aiming to provide insights into predicting and controlling indoor PM_2.5_ concentrations via ventilation system operation. The impacts of filter efficiency, ventilation flow rates, and outdoor PM_2.5_ concentrations on indoor air quality were assessed using mathematical modeling and experimentation. The results revealed that using filters rated MERV 13 or higher was more advantageous than using lower rated ones. Additionally, the study developed a mass balance equation for indoor PM_2.5_ and demonstrated that this equation accurately matched the measured values. These findings enable the development of effective strategies for maintaining indoor air quality and minimizing exposure to fine particulate matter in residential houses.

## Figures and Tables

**Table 1:** Test house information for substitution in Equation (2).

Apartment	Year of Completion (Year)	Exclusive Area (m^2^)	Test Volume, V (m^3^)	Mechanical Ventilation			ACH50 (1/h)	$\mathrm{Initial}\;\mathrm{PM}{2.5_{\;}}^{1},\;C_{0}$ (μg/m^3^)	$\mathrm{Average}\;\mathrm{Outdoor}\;{\mathrm{PM}}_{2.5},\;C_{out}$ (μg/m^3^)	$\mathrm{Deposition}\;\mathrm{Rate},\;\dot{S}$ (1/h)	$\mathrm{Penetration}\;\mathrm{of}\;\mathrm{Wall},\;P_{inf}$ (%)
				$\mathrm{Supply}\;\mathrm{Air}\;\mathrm{Flow}\;\mathrm{Rate},\;Q_{SA}$ (m^3^/h)	$\mathrm{Exhaust}\;\mathrm{Air}\;\mathrm{Flow}\;\mathrm{Rate},\;Q_{RA}$ (m^3^/h)	$\mathrm{Collection}\;\mathrm{Efficiency},\;1-P_{MV}$ (%)					
A	2018	72	84	44	56	90	2.1	43.2	30	0.1	90
B	2018	75	72	79	50	52	2.1	25	31	0.1	90
C	2015	70	72	80	0	72	2.1	29.9	36	0.1	90
D	2017	75	85	50	39	20	2.1	12.4	16	0.1	90
E	2013	85	85	167	142	41	2.1	11.2	16	0.1	90
F	2017	85	85	70	35	60	2.1	36.1	50	0.1	90
G	2016	85	85	138	167	92	2.1	28.1	31	0.1	90

^1^ PM_2.5_, fine particulate matter.

## Data Availability

Data are contained within the article.
